# Supplementary material for: Multiple instance fine-mapping: Predicting causal regulatory variants with a deep sequence model
Source: PLoS Genet. 2026 Jun 29;22(6):e1012208. doi: 10.1371/journal.pgen.1012208 (PMC13354094; doi:10.1371/journal.pgen.1012208)
Supplement: S1 Appendix — Fig A in S1 Appendix Ancestry-stratified performance comparison of polygenic scores (PGS) created with MIFM and baseline methods on 5 non-European ancestries and 20 phenotypes. We counted the number of scenarios where MIFM would perform better than a baseline (in green), worse (in red), or not significantly different (in gray). Fig B in S1 Appendix Per-trait performance comparison of PGS created with MIFM and baseline methods on 5 non-European ancestries and 20 phenotypes. We counted the number of scenarios where MIFM would perform better than a baseline (in green), worse (in red), or not significantly different (in gray). Traits are sorted by the net difference in scenarios where MIFM was better, i.e., #Better - #Worse. Fig C in S1 Appendix Performance comparison of top-5 variants-per-block PGS created with MIFM and 12 baseline methods on 5 non-European ancestries and 20 traits. We created PGS using results from 20 genome-wide association studies (GWASs) performed on European samples and evaluated them on 5 non-European samples, yielding 100 test scenarios per model. For each baseline, we counted the num- ber of scenarios where MIFM would perform better than the baseline (in green), worse (in red), or not significantly different (in gray). Fig D in S1 Appendix Performance comparison of top-10 variants-per-block PGS created with MIFM and 12 baseline methods on 5 non-European ancestries and 20 traits. We created PGS using results from 20 GWASs performed on European samples and evaluated them on 5 non-European samples, yielding 100 test scenarios per model. For each baseline, we counted the number of scenarios where MIFM would perform better than the baseline (in green), worse (in red), or not significantly different (in gray). Fig E in S1 Appendix Mean performance measured by R2 of top-5 variants-per-block PGS created with MIFM and 12 baseline methods on 5 non-European ancestries and 20 traits. We created PGS using results from 20 GWASs performed on European samples [file pgen.1012208.s001.pdf]

List of Figures

A **Ancestry-stratified performance comparison of polygenic scores (PGS) created with MIFM and baseline methods on 5 non-European ancestries and 20 phenotypes.** We counted the number of scenarios where MIFM would perform better than a baseline (in green), worse (in red), or not significantly different (in gray). 2

B **Per-trait performance comparison of PGS created with MIFM and baseline methods on 5 non-European ancestries and 20 phenotypes.** We counted the number of scenarios where MIFM would perform better than a baseline (in green), worse (in red), or not significantly different (in gray). Traits are sorted by the net difference in scenarios where MIFM was better, i.e., *#Better - #Worse*. 2

C **Performance comparison of top-5 variants-per-block PGS created with MIFM and 12 baseline methods on 5 non-European ancestries and 20 traits.** We created PGS using results from 20 genome-wide association studies (GWASs) performed on European samples and evaluated them on 5 non-European samples, yielding 100 test scenarios per model. For each baseline, we counted the number of scenarios where MIFM would perform better than the baseline (in green), worse (in red), or not significantly different (in gray). 3

D **Performance comparison of top-10 variants-per-block PGS created with MIFM and 12 baseline methods on 5 non-European ancestries and 20 traits.** We created PGS using results from 20 GWASs performed on European samples and evaluated them on 5 non-European samples, yielding 100 test scenarios per model. For each baseline, we counted the number of scenarios where MIFM would perform better than the baseline (in green), worse (in red), or not significantly different (in gray). 3

E **Mean performance measured by R^2 of top-5 variants-per-block PGS created with MIFM and 12 baseline methods on 5 non-European ancestries and 20 traits.** We created PGS using results from 20 GWASs performed on European samples and evaluated them on 5 non-European samples, yielding 100 test scenarios per model. 4

F **Mean performance measured by R^2 of top-10 variants-per-block PGS created with MIFM and 12 baseline methods on 5 non-European ancestries and 20 traits.** We created PGS using results from 20 GWASs performed on European samples and evaluated them on 5 non-European samples, yielding 100 test scenarios per model. 5

List of Tables

A Enrichment of enhancer regions in repressed-enhancer regions prioritized by MIFM. 6

B Enrichment of enhancer regions in repressed regions prioritized by MIFM. 6

C Enrichment of silencer elements in repressed-enhancer regions prioritized by MIFM. 6

D Enrichment of silencers in repressed regions prioritized by MIFM. 6

E Enrichment of silencers in enhancer regions prioritized by MIFM. 7

F **Transcription factor motifs matched to patterns identifed in MIFM using Transcription-Factor Motif Discovery from Importance Scores (TF-MoDISco).** *Pattern type* denotes whether a TF-MoDISco pattern contributes positively or negatively to MIFM predictions. *TF motif* denotes the name of the transcription factor. *No. seqlets* – the total number of TF-MoDISco seqlets matching the given transcription factor (TF) motif. *No. patterns* – the total number of different TF-MoDISco patterns matching the given TF motif. 7

1 Evaluation of polygenic scores

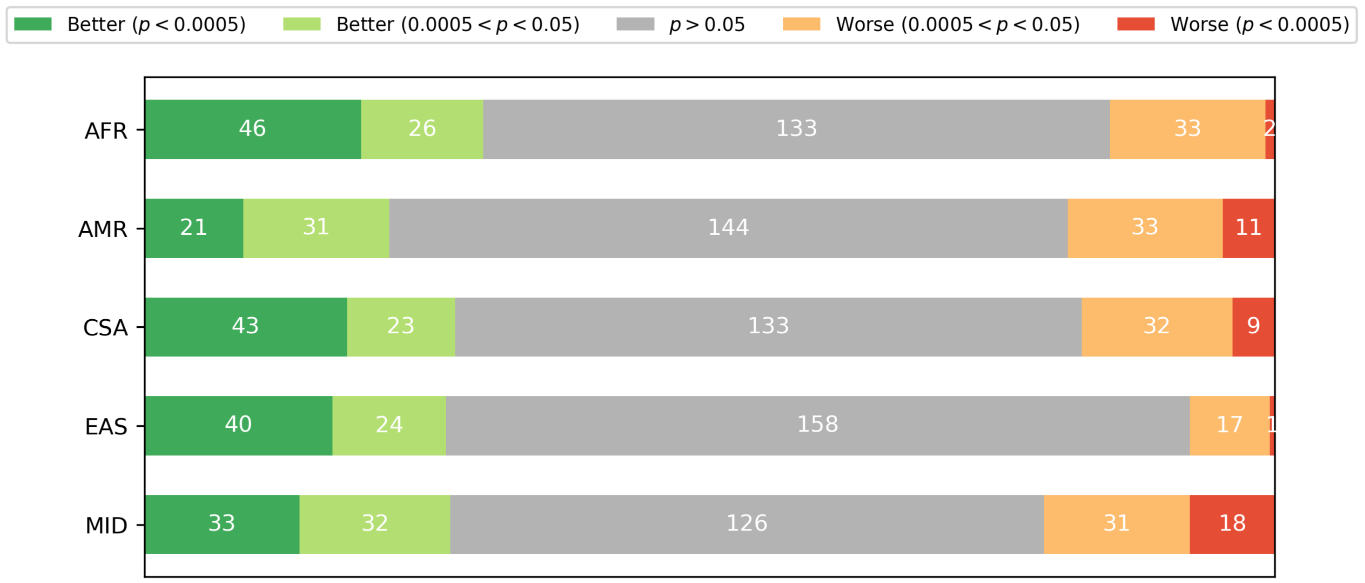

Fig A: **Ancestry-stratified performance comparison of PGS created with MIFM and baseline methods on 5 non-European ancestries and 20 phenotypes.** We counted the number of scenarios where MIFM would perform better than a baseline (in green), worse (in red), or not significantly different (in gray).

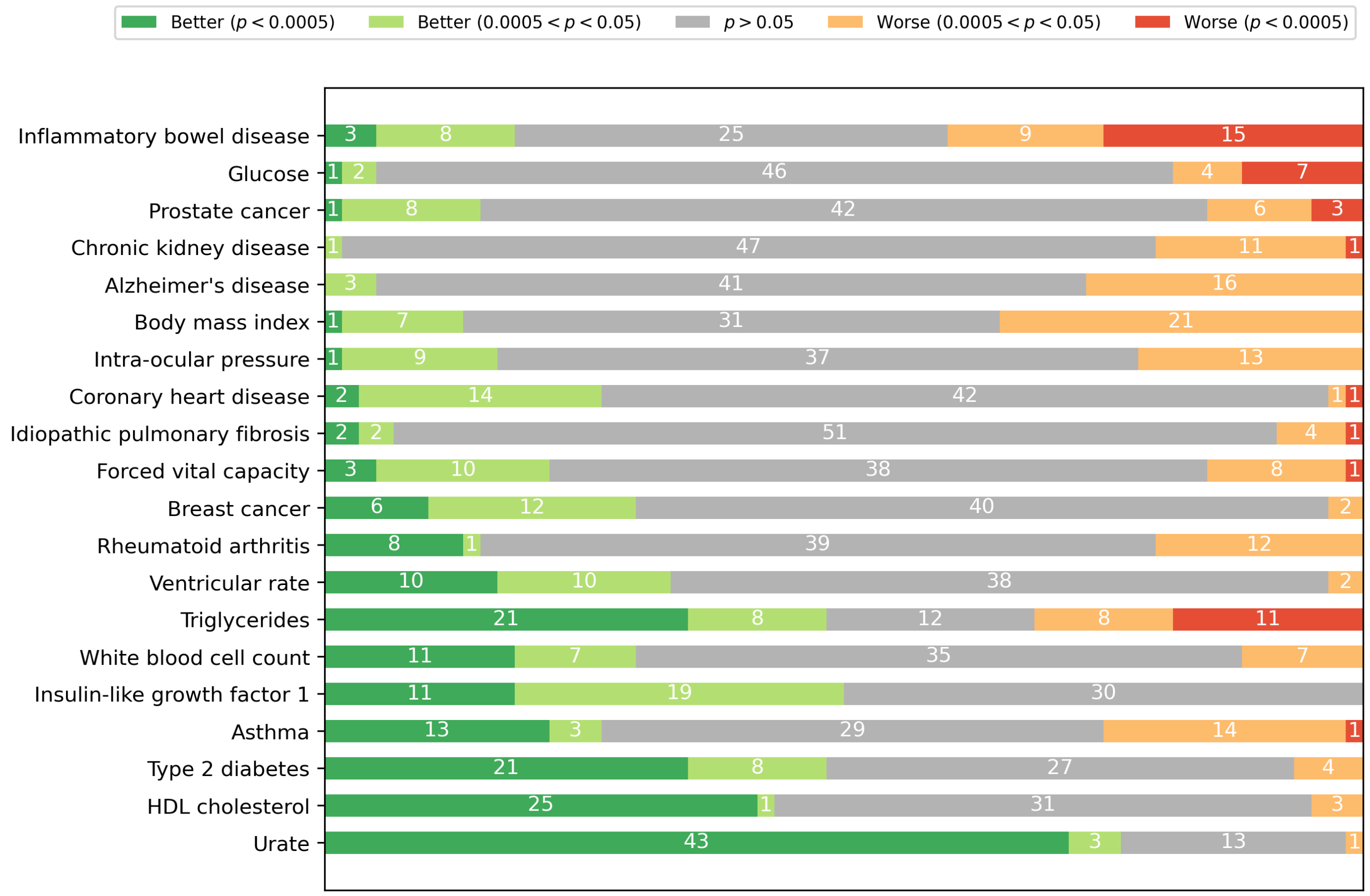

Fig B: **Per-trait performance comparison of PGS created with MIFM and baseline methods on 5 non-European ancestries and 20 phenotypes.** We counted the number of scenarios where MIFM would perform better than a baseline (in green), worse (in red), or not significantly different (in gray). Traits are sorted by the net difference in scenarios where MIFM was better, i.e., *#Better - #Worse*.

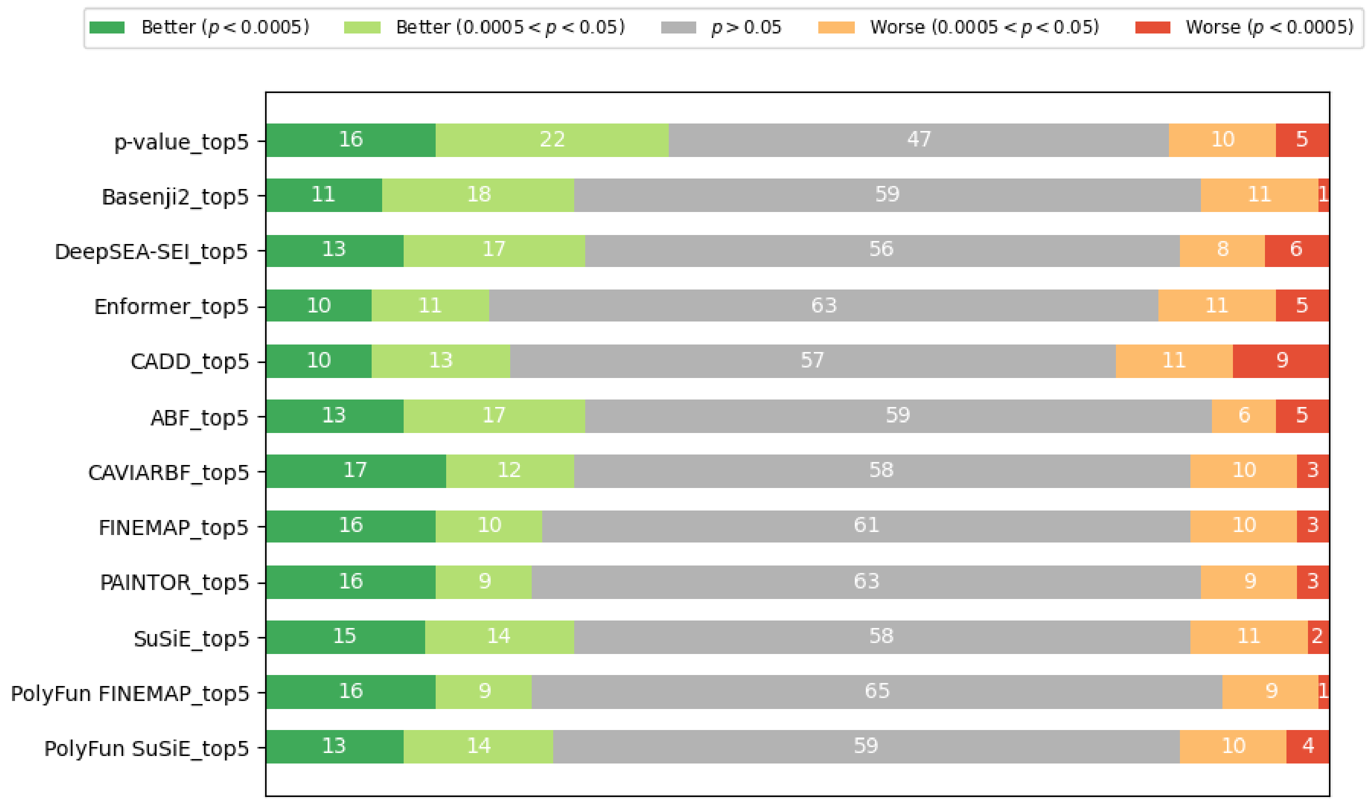

Fig C: **Performance comparison of top-5 variants-per-block PGS created with MIFM and 12 baseline methods on 5 non-European ancestries and 20 traits.** We created PGS using results from 20 GWASs performed on European samples and evaluated them on 5 non-European samples, yielding 100 test scenarios per model. For each baseline, we counted the number of scenarios where MIFM would perform better than the baseline (in green), worse (in red), or not significantly different (in gray).

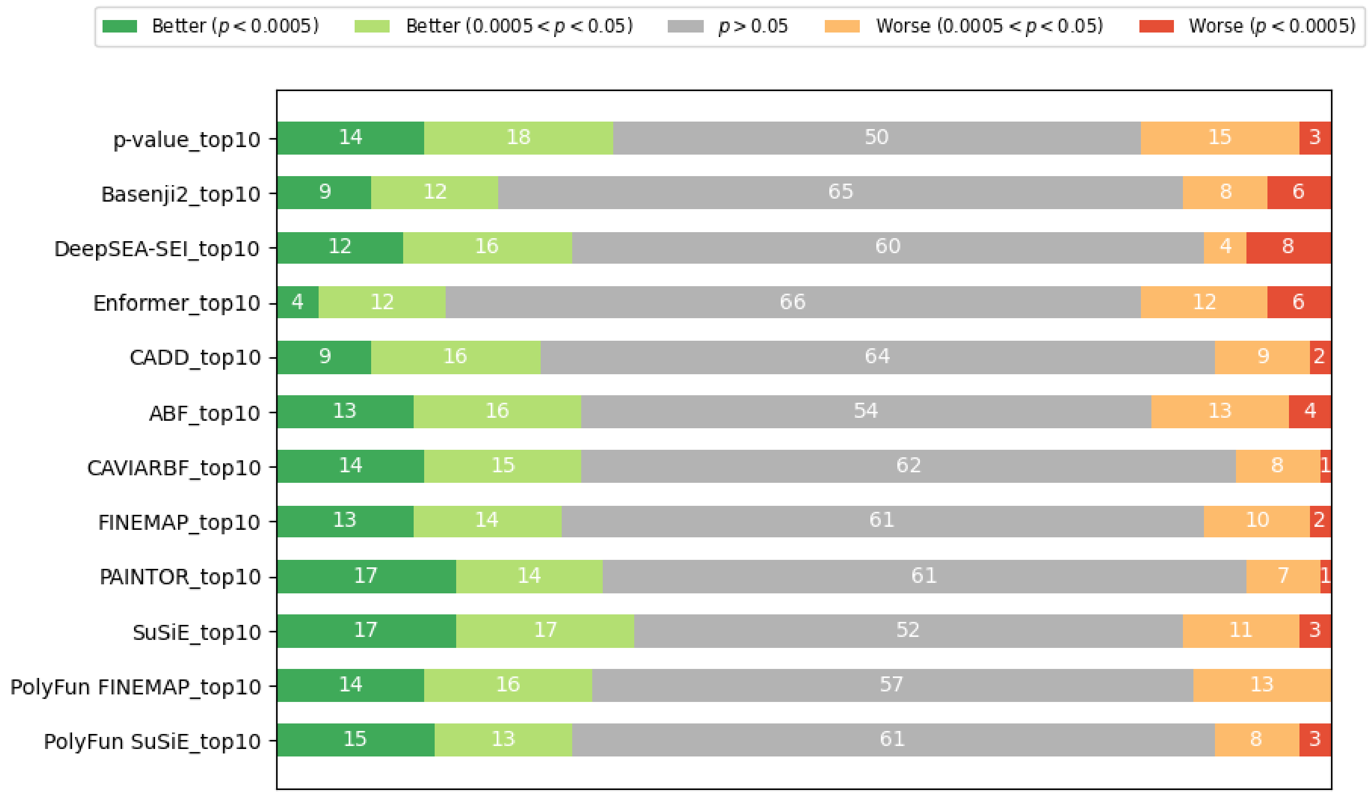

Fig D: **Performance comparison of top-10 variants-per-block PGS created with MIFM and 12 baseline methods on 5 non-European ancestries and 20 traits.** We created PGS using results from 20 GWASs performed on European samples and evaluated them on 5 non-European samples, yielding 100 test scenarios per model. For each baseline, we counted the number of scenarios where MIFM would perform better than the baseline (in green), worse (in red), or not significantly different (in gray).

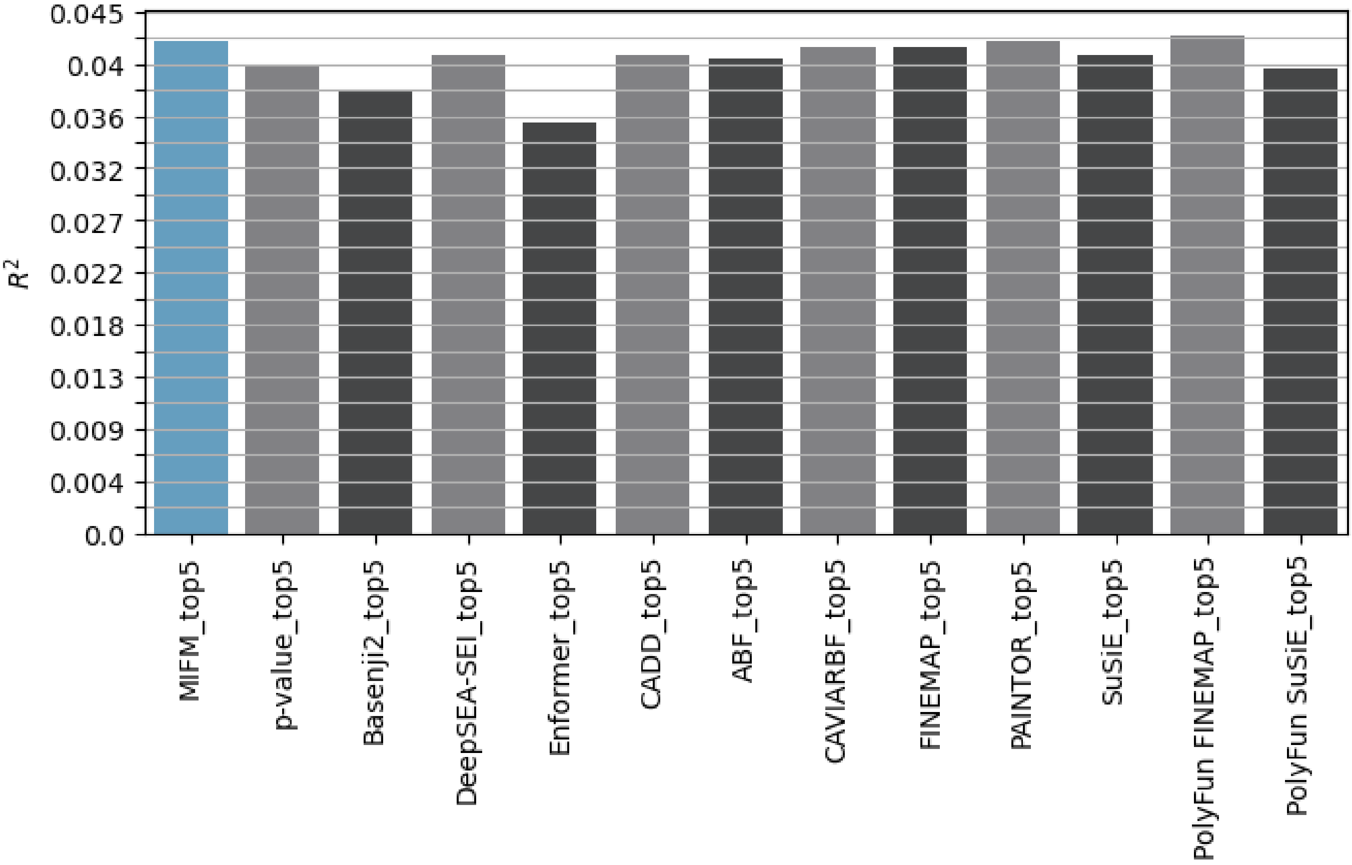

Fig E: **Mean performance measured by R^2 of top-5 variants-per-block PGS created with MIFM and 12 baseline methods on 5 non-European ancestries and 20 traits.** We created PGS using results from 20 GWASs performed on European samples and evaluated them on 5 non-European samples, yielding 100 test scenarios per model.

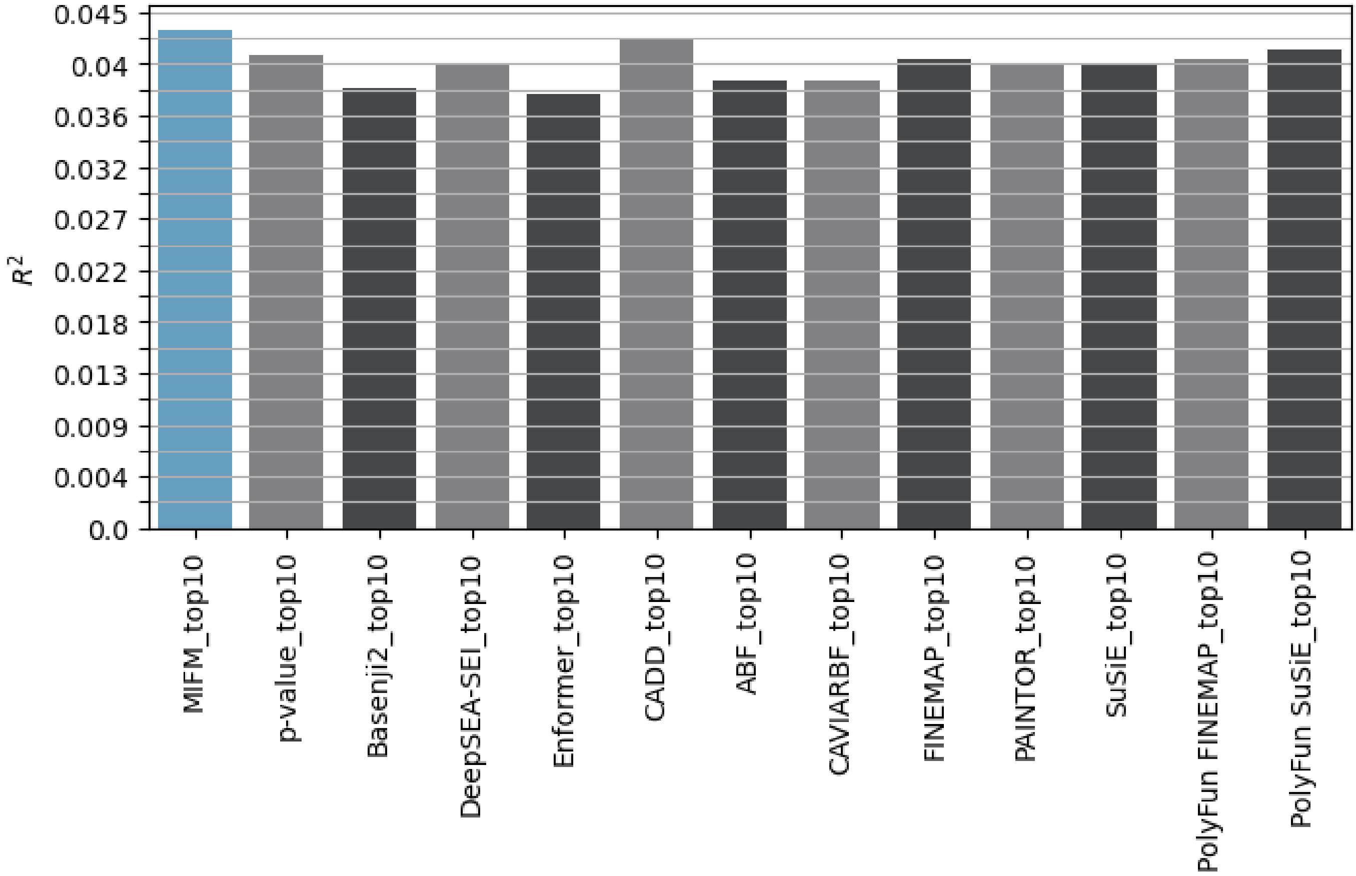

Fig F: **Mean performance measured by R^2 of top-10 variants-per-block PGS created with MIFM and 12 baseline methods on 5 non-European ancestries and 20 traits.** We created PGS using results from 20 GWASs performed on European samples and evaluated them on 5 non-European samples, yielding 100 test scenarios per model.

2 Enrichment of regulatory classes

Table A: Enrichment of enhancer regions in repressed-enhancer regions prioritized by MIFM.

MIFM score quantile	OR	p-value
0.1	1.02	9.88e-02
0.2	1.03	7.06e-03
0.3	1.05	6.72e-05
0.4	1.06	6.67e-07
0.5	1.08	3.53e-08
0.6	1.09	1.04e-08
0.7	1.09	2.68e-08
0.8	1.09	1.88e-06
0.9	1.10	1.17e-04

Table B: Enrichment of enhancer regions in repressed regions prioritized by MIFM.

MIFM score quantile	OR	p-value
0.1	1.01	2.27e-05
0.2	1.02	8.80e-12
0.3	1.03	1.01e-19
0.4	1.03	1.71e-26
0.5	1.04	1.28e-32
0.6	1.04	1.16e-37
0.7	1.05	1.90e-44
0.8	1.06	1.50e-45
0.9	1.08	8.19e-40

Table C: Enrichment of silencer elements in repressed-enhancer regions prioritized by MIFM.

MIFM score quantile	OR	p-value
0.1	1.00	0.96
0.2	1.00	0.76
0.3	1.00	0.81
0.4	1.00	0.93
0.5	1.00	0.86
0.6	1.00	0.94
0.7	1.01	0.73
0.8	1.00	0.86
0.9	0.97	0.21

Table D: Enrichment of silencers in repressed regions prioritized by MIFM.

MIFM score quantile	OR	p-value
0.1	1.01	1.21e-02
0.2	1.02	3.29e-06
0.3	1.02	1.92e-09
0.4	1.03	3.53e-12
0.5	1.03	7.80e-15
0.6	1.04	4.35e-16
0.7	1.05	5.47e-21
0.8	1.06	4.21e-23
0.9	1.07	6.93e-22

Table E: Enrichment of silencers in enhancer regions prioritized by MIFM.

MIFM score quantile	OR	p-value
0.1	1.01	2.77e-02
0.2	1.02	3.20e-05
0.3	1.02	4.47e-08
0.4	1.03	1.05e-10
0.5	1.03	1.31e-14
0.6	1.04	1.44e-16
0.7	1.05	1.70e-18
0.8	1.06	3.81e-20
0.9	1.07	6.63e-16

3 Syntax analysis of MIFM

Table F: **Transcription factor motifs matched to patterns identifed in MIFM using TF-MoDISco.** *Pattern type* denotes whether a TF-MoDISco pattern contributes positively or negatively to MIFM predictions. *TF motif* denotes the name of the transcription factor. *No. seqlets* – the total number of TF-MoDISco seqlets matching the given TF motif. *No. patterns* – the total number of different TF-MoDISco patterns matching the given TF motif.

Pattern type	TF name	No. seqlets	No. patterns
Positive	LMX1A	21464	9
	PO3F3	14847	8
	CPEB1	14382	6
	HXC10	13213	5
	ARI3A	12479	5
	FOXG1	4767	2
	LMX1B	4392	2
	TEAD4	3516	3
	FOXD2	2744	1
	PO3F4	2136	1
	PO4F3	2082	1
	PO4F1	2082	1
	ZN467	1867	1
	MAZ	1867	1
	ZN263	1867	1
	ZN341	1867	1
	ZN770	1439	2
	TEAD1	1435	1
	ZBT18	909	1
	PAX5	563	1
	ZN121	563	1
	ZN770	468	1
	ZSC22	468	1
	ZFX	468	1
	THA	196	1
	RXRA	196	1

Pattern type	TF name	No. seqlets	No. patterns
Negative	CPEB1	27434	31
	LMX1A	22784	15
	ARI3A	15405	12
	PO3F3	15046	9
	FOXL1	12794	18
	HXC10	12771	11
	FOXG1	12287	16
	FOXD2	8653	7
	PRDM6	7969	15
	FOXJ3	6334	7
	ZN467	2959	2
	VEZF1	2105	1
	PATZ1	2105	1
	SRBP2	2105	1
	GSX1	1998	1
	IRF1	1177	1
	ZN121	998	2
	PAX5	894	1
	RXRA	876	1
	ELF3	876	1
	EHF	876	1
	PATZ1	876	1
	KLF15	854	1
	TBX15	854	1
	MAZ	854	1
	ZFP82	773	1
	P5F1B	617	1
	PITX2	350	1
	ANDR	347	1
	NFAC1	280	1
	BC11A	280	1
	ZN770	215	1
	ZN770	215	1
	ZSC22	215	1
	ZFP28	115	1
	FOXK1	86	1
	ZN713	52	1
