# Supplementary material for: Multiple instance fine-mapping: Predicting causal regulatory variants with a deep sequence model
Source: PLoS Genet. 2026 Jun 29;22(6):e1012208. doi: 10.1371/journal.pgen.1012208 (PMC13354094; doi:10.1371/journal.pgen.1012208)
Supplement: Attachment — Submitted filename: 6251e89e-8bc5-4e1c-ae6f-a010c8f4c013_Rebuttal_letter.pdf [file pgen.1012208.s005.pdf]

Rebuttal letter

Dear PLOS Genetics Editors,

The decision letter for the previous version of the manuscript stated:

"However, we will be willing to consider a substantial revision that can demonstrate MIFM performing better than other fine-mapping methods through constructing PRSs in transferring across ancestries. In particular, it will be informative to see the the actual differences in prediction Rsq achieved by the PRSs between MIFM and other fine-mapping methods. There are also many other computational methods to annotate SNPs such as CADD, DeepSEA and Basenji. It will be reasonable to compare MIFM with these existing methods."

We have included the additional baselines mentioned: CADD, Basenji2, and the latest version of DeepSEA (DeepSEA-SEI), included a plot of the achieved R-squared scores for all methods in the main manuscript (Figure 2), and included a table with R-squared scores for all model-trait-ancestry combinations in the supplementary materials. We note that MIFM still performs better than the additional baselines, both in terms of the number of scenarios (previously included evaluation) as well as in terms of the R-squared coefficients.

We are eager to answer any other questions or provide additional analyses.

Kind regards,

Alexander Rakowski
