# Supplementary material for: Multiple instance fine-mapping: Predicting causal regulatory variants with a deep sequence model
Source: PLoS Genet. 2026 Jun 29;22(6):e1012208. doi: 10.1371/journal.pgen.1012208 (PMC13354094; doi:10.1371/journal.pgen.1012208)
Supplement: Attachment — Submitted filename: 16_03_2026_Response_to_Reviewers.pdf [file pgen.1012208.s006.pdf]

Response to Reviewers

Reviewer #1:

1. *Since the 20 traits (10 continuous, 10 binary) GWAS that formed the evaluation set were also selected from CAUSALdb2, have those loci/signals been also included in the training corpus before and, therefore, already seen by the model (e.g. vs other benchmarking models that have not been trained on these sequences)? If yes, could that lead to inflated results? For a more objective evaluation of generalization ability, the author is recommended to leave the GWAS associated with the 20 traits out during pretraining and compute the PGS of these GWAS during evaluation.*

We have now replaced the PGS results with a model trained without the 20 traits. The new model still outperformed the baselines, however it had a larger number of scenarios where it performed significantly worse than a baseline.

As a side-note, as described at the end of Section 5.2., in a practical scenario, one can still use the results from a GWAS of interest (where fine-mapping is to be performed) to train the model, as we only use weak labels - we never have access to the ground-truth labels of variant causality, and use GWAS-significance as the proxy weak labels.

2. *How exactly were the PGS constructed? I understand the part that the authors take the top SNP per block, but what about the allele orientation and per-SNP weight (e.g. whether the authors used the model-predicted score as weight here, or the original GWAS beta, or other weights)? The allele orientation also matters as inverting the sign changes the score and results. I did not find details in the manuscript and recommend that the authors include explicit formula explaining this step.*

We have used the original GWAS effect sizes, allele orientations, etc. Model scores are just used to select the top variant per-block. We have updated the manuscript to reflect this information.

***3.** Certain baseline methods – e.g. SuSiE, FINEMAP, CAVIARBF, PolyFun FINEMAP etc – by design outputs continuous posterior inclusion probability (for each SNP) and credible sets (for each effect component), and therefore favors "outputting >1 causal SNP per locus" and carrying forward "uncertainty" (probabilistic, rather than forcing a single winner). Forcing to choose the highest fine-mapping annotation score from each block based on their annotations would tend to put them in disadvantage in comparison (e.g. if there are 5 SNPs in tight LD, these methods tend to "spread" the probability, and "pick-one" rule may pick the wrong one). To ensure a fair comparison it is recommended that the authors adopt a multi-variant selection (i.e. top-k per block, instead of top 1) and build PGS with a common weighting scheme.*

We have included two additional settings of the PGS evaluation, taking the top 5, and top 10 variants per block with a common weighting scheme. The corresponding figures are included in the supplementary material. The probabilistic methods performed similarly in terms of #better/#worse scenarios, but improved visibly in terms of the total R2 scores, with PolyFun FINEMAP outperforming MIFM in the top-5 variants per-block setting.

***4.** Other more advanced, pretrained DNN models (e.g. Enformer) simultaneously predict for many tasks/tracks, across different cell types, tissues and assays. For example, Enformer outputs predictions for 5,313 human tracks (2,131 TF ChIP-Seq, 1,860 histone modification ChIP–seq, 684 DNase-seq or ATAC-seq, and 638 CAGE tracks). The authors mentioned that they "calculated the annotation scores as the maximum differences in predictions for the alternative versus reference allele over all model outputs", which does not seem to make sense here as most traits are tissue/cell/context specific – the maximum value picked here may not reflect the correct biological context. Moreover, different tracks tend to have different range of predictions, and simply choosing the max may not necessarily indicate the most relevant biology. A more carefully designed scoring scheme should be adopted here, again to ensure a fair comparison across models.*

We have now re-scored variants for Basenji2/Enformer the following way. For both models, we have computed the mean and standard deviation of each prediction track using 1,000 Genomes common variants, and used these to normalize each prediction track to a z-score, enabling a more direct comparison of their values,

as they should now lie in a similar range. We have updated the text and the PGS results accordingly.

5. *The negatives are drawn from common variants (MAF≥1%) that are >128 bp from positives. This selection might induce frequency and genomic-context mismatches (and nontrivial label noise), which could bias the learned decision boundary. For example, the sequence length is 512 (512/2 = 256 > 128), which means the negative still contains a part of positive signal in its input window. Would that affect the performance? Even if the positive base isn't inside the negative's window, a negative can still be in high LD with a positive. Meanwhile, if positives and negatives don't share the same MAF distribution within each locus/genome-wide, the model might exploit frequency-correlated sequence features (e.g., CpG depletion, constraint) as shortcuts and end up learning "is this sequence typical of common variants?".*

We have matched the negative examples by CpG content to the positive examples. It is true that a negative sequence might contain a positive variant, but this is in fact desired, as we are interested in making variant-level predictions (i.e., at the central position of the sequence), and not sequence-level predictions. Negative examples potentially containing the positive variant at the sides of the sequence, or a variant in the middle of the sequence in high LD with a causal variant still constitute non-causal variants, and should be predicted as such - ideally, only sequences where the causal variant is in the exact middle should be predicted as causal.

6*. Do the different bag sizes also affect the results? With max pooling, larger bags might dominate gradients. Also, the expected maximum grows with bag size, resulting in a priori higher likelihood to have a high bag score. Meanwhile, the positives are multi-SNP bags while negatives contain only one SNP, would that also bias the model to learn "larger bag sizes (more SNPs) in a bag → positive"? It is advisable that the authors report performance vs. different bag size, and also redesign the negative bags such that they have similar bag size.*

We understand the concern about large bags "dominating" gradients, however, we would want to clarify: by construction, each positive bag has a corresponding training label of 1, and each negative a label of 0. By using max pooling, within a

positive bag, the gradient is propagated from the (single) sequence in the bag with the highest predicted score (in the case of negative bags a negative gradient is propagated for each negative example). However, each whole bag is weighted equally, regardless of their size. Mechanistically, the network makes a separate prediction for each example in the bag, and these are then passed through the max pooling, but the network has no way of inferring the actual size of the bag, and thus no way of biasing the predictions based on the bag size.

Reviewer #2

1. *Section 3.5 in the Results ("Syntax analysis of a MIFM trained model") starts with what the authors did, but it is not clear why they are doing that. This paragraph should start with a sentence saying what is the question that the section/paragraph is trying to answer. The authors directly jump into what they did "We analyzed..."*

We have updated the manuscript accordingly.

2. ** In Methods, line 227, in the text "... be the p-value for the k-th variant" maybe you meant "j-th variant"? (If I'm following correctly).*

Yes, that is correct. We have updated the manuscript accordingly.

3. *In Methods, equations 4 and 5, when you define positive and negative bags, how do you handle cases where variants are borderline with respect to the threshold? Shouldn't you use a larger gap to ensure non-significant results? In the current definition, with a threshold of 0.05 (just as an example) a variant with p-value 0.04999 would be positive, and one with p-value 0.050001 would be negative. Could this be a potential problem?*

We agree this is a relevant concern. Equations 4 and 5 are meant as a formal description of the setting. In practice, one can employ any arbitrary filtering of the negatives, e.g., with a larger p-value gap, based on functional annotations of genetic regions, etc., as long as the variants do not invalidate the thresholds from Eqs. 4-5, i.e., no negative variant should exceed the significance threshold and vice versa. Caution should be taken when taking the "non-gapped" threshold (e.g., neg > 0.05, pos < 0.05) when a small number of results from well-powered GWAS are available per variant, which could indeed lead to the presence of causal variants in negative bags.

4. *In Methods, under "Dataset and model training" could the authors explain more the reason for having a token "V"? Is it correct that, per bag, this indicates the variant of interest?*

It indicates the variant of interest per-example(/sequence) - a bag can contain multiple examples with a "V" token. The rationale was to force the model to not only rely on "general" sequence features, but also focus on the variant itself. This way we can employ data augmentations in the form of random sequence shifts - the "V" token would be then shifted from the center of the sequence, and construct negative examples with negative variants close to a putative causal variant: e.g., for a (hypothetical) sequence AAATAAA with the causal variant being only the 4th position "T>A", the model should distinguish between the inputs AAAVAAA (potentially causal) and AAVTAAA/AVATAAA/VAATAAA/… (negative).

5. *There is a GitHub repository (https://github.com/HealthML/multiple-instance-fine-mapping) with the method and examples on how to make predictions using a pre-trained model. However, the code for reproducing the figures/tables is not available.*

We have updated the repository accordingly.
