# Supplementary material for: Multiple instance fine-mapping: Predicting causal regulatory variants with a deep sequence model
Source: PLoS Genet. 2026 Jun 29;22(6):e1012208. doi: 10.1371/journal.pgen.1012208 (PMC13354094; doi:10.1371/journal.pgen.1012208)
Supplement: Peer review history

# Author comment: Author response to Decision Letter 0

**DOI:** 10.1371/journal.pgen.1012208.r001

11 Jul 2025

Attachment Submitted filename: 6251e89e-8bc5-4e1c-ae6f-a010c8f4c013_Rebuttal_letter.pdf

# Peer review: Decision Letter 0

**DOI:** 10.1371/journal.pgen.1012208.r002

**Authors:** Heather Cordell, Xiaofeng Zhu

15 Jan 2026

PGENETICS-D-25-00787

Multiple instance fine-mapping: predicting causal regulatory variants with a deep sequence model

PLOS Genetics

Dear Dr. Rakowski,

Thank you for submitting your manuscript to PLOS Genetics. After careful consideration, we feel that it has merit but does not fully meet PLOS Genetics's publication criteria as it currently stands. Therefore, we invite you to submit a revised version of the manuscript that addresses the points raised during the review process.

Please submit your revised manuscript by Mar 16 2026 11:59PM. If you will need more time than this to complete your revisions, please reply to this message or contact the journal office at plosgenetics@plos.org. When you're ready to submit your revision, log on to https://www.editorialmanager.com/pgenetics/ and select the 'Submissions Needing Revision' folder to locate your manuscript file.

Please include the following items when submitting your revised manuscript:

* A letter that responds to each point raised by the editor and reviewer(s). You should upload this letter as a separate file labeled 'Response to Reviewers'. This file does not need to include responses to any formatting updates and technical items listed in the 'Journal Requirements' section below.

* A marked-up copy of your manuscript that highlights changes made to the original version. You should upload this as a separate file labeled 'Revised Manuscript with Track Changes'.

* An unmarked version of your revised paper without tracked changes. You should upload this as a separate file labeled 'Manuscript'.

If you would like to make changes to your financial disclosure, competing interests statement, or data availability statement, please make these updates within the submission form at the time of resubmission. Guidelines for resubmitting your figure files are available below the reviewer comments at the end of this letter.

We look forward to receiving your revised manuscript.

Kind regards,

Heather J Cordell

Academic Editor

PLOS Genetics

Xiaofeng Zhu

Section Editor

PLOS Genetics

Aimée Dudley

Editor-in-Chief

PLOS Genetics

Anne Goriely

Editor-in-Chief

PLOS Genetics

**Journal Requirements:**

1) We ask that a manuscript source file is provided at Revision. Please upload your manuscript file as a .doc, .docx, .rtf or .tex. If you are providing a .tex file, please upload it under the item type u2018LaTeX Source Fileu2019 and leave your .pdf version as the item type u2018Manuscriptu2019.

2) Your manuscript is missing the following sections: Verification and Comparison, Applications, Acknowledgements, and Supplementary Information. Please ensure that your article adheres to the standard Methods article layout and order of Abstract, Author Summary, Introduction, Description of the Method, Verification and Comparison, Applications, Discussion, Acknowledgements, References, and Supplementary Information. For details on what each section should contain, see our Methods article guidelines:

https://journals.plos.org/plosgenetics/s/submission-guidelines#loc-manuscript-organization.

3) Please upload all main figures as separate Figure files in .tif or .eps format. For more information about how to convert and format your figure files please see our guidelines:

https://journals.plos.org/plosgenetics/s/figures

4) We have noticed that you have uploaded Supporting Information files, but you have not included a list of legends. Please add a full list of legends for your Supporting Information files after the references list.

5) Please amend your detailed Financial Disclosure statement. This is published with the article. It must therefore be completed in full sentences and contain the exact wording you wish to be published.

State the initials, alongside each funding source, of each author to receive each grant. For example: "This work was supported by the National Institutes of Health (####### to AM; ###### to CJ) and the National Science Foundation (###### to AM)."

State what role the funders took in the study. If the funders had no role in your study, please state: "The funders had no role in study design, data collection and analysis, decision to publish, or preparation of the manuscript.".

If you did not receive any funding for this study, please simply state: u201cThe authors received no specific funding for this work.u201d

**Reviewers' comments:**

Reviewer's Responses to Questions

**Comments to the Authors:**

**Please note here if the review is uploaded as an attachment.**

Reviewer #1: General comments

In this work, the authors present MIFM, a deep sequence model trained in a multiple-instance learning (MIL) framework where LD-defined loci are treated as bags and individual SNPs as instances. Using weak bag-level labels aggregated from CAUSALdb2, the model learns an instance-level “causality” score and is then used to prioritize variants for (i) cross-ancestry PGS construction (select-one-per-block paradigm), (ii) conditional analyses in moderately sized cohorts, and (iii) functional enrichment/motif analyses. Conceptually, the MIL-on-LD formulation is elegant and, to my knowledge, novel among sequence-based approaches. However, several aspects of the evaluation and methodological specification, in my view, need to be further addressed to ensure fair comparisons and showcase the full reliability of the results.

Specific comments

1. Since the 20 traits (10 continuous, 10 binary) GWAS that formed the evaluation set were also selected from CAUSALdb2, have those loci/signals been also included in the training corpus before and, therefore, already seen by the model (e.g. vs other benchmarking models that have not been trained on these sequences)? If yes, could that lead to inflated results? For a more objective evaluation of generalization ability, the author is recommended to leave the GWAS associated with the 20 traits out during pretraining and compute the PGS of these GWAS during evaluation.

2. How exactly were the PGS constructed? I understand the part that the authors take the top SNP per block, but what about the allele orientation and per-SNP weight (e.g. whether the authors used the model-predicted score as weight here, or the original GWAS beta, or other weights)? The allele orientation also matters as inverting the sign changes the score and results. I did not find details in the manuscript and recommend that the authors include explicit formula explaining this step.

3. Certain baseline methods – e.g. SuSiE, FINEMAP, CAVIARBF, PolyFun FINEMAP etc – by design outputs continuous posterior inclusion probability (for each SNP) and credible sets (for each effect component), and therefore favors “outputting >1 causal SNP per locus” and carrying forward “uncertainty” (probabilistic, rather than forcing a single winner). Forcing to choose the highest fine-mapping annotation score from each block based on their annotations would tend to put them in disadvantage in comparison (e.g. if there are 5 SNPs in tight LD, these methods tend to “spread” the probability, and “pick-one” rule may pick the wrong one). To ensure a fair comparison it is recommended that the authors adopt a multi-variant selection (i.e. top-k per block, instead of top 1) and build PGS with a common weighting scheme.

4. Other more advanced, pretrained DNN models (e.g. Enformer) simultaneously predict for many tasks/tracks, across different cell types, tissues and assays. For example, Enformer outputs predictions for 5,313 human tracks (2,131 TF ChIP-Seq, 1,860 histone modification ChIP–seq, 684 DNase-seq or ATAC-seq, and 638 CAGE tracks). The authors mentioned that they “calculated the annotation scores as the maximum differences in predictions for the alternative versus reference allele over all model outputs”, which does not seem to make sense here as most traits are tissue/cell/context specific – the maximum value picked here may not reflect the correct biological context. Moreover, different tracks tend to have different range of predictions, and simply choosing the max may not necessarily indicate the most relevant biology. A more carefully designed scoring scheme should be adopted here, again to ensure a fair comparison across models.

5. The negatives are drawn from common variants (MAF≥1%) that are >128 bp from positives. This selection might induce frequency and genomic-context mismatches (and nontrivial label noise), which could bias the learned decision boundary. For example, the sequence length is 512 (512/2 = 256 > 128), which means the negative still contains a part of positive signal in its input window. Would that affect the performance? Even if the positive base isn’t inside the negative’s window, a negative can still be in high LD with a positive. Meanwhile, if positives and negatives don’t share the same MAF distribution within each locus/genome-wide, the model might exploit frequency-correlated sequence features (e.g., CpG depletion, constraint) as shortcuts and end up learning “is this sequence typical of common variants?”.

6. Do the different bag sizes also affect the results? With max pooling, larger bags might dominate gradients. Also, the expected maximum grows with bag size, resulting in a priori higher likelihood to have a high bag score. Meanwhile, the positives are multi-SNP bags while negatives contain only one SNP, would that also bias the model to learn “larger bag sizes (more SNPs) in a bag -> positive”? It is advisable that the authors report performance vs. different bag size, and also redesign the negative bags such that they have similar bag size.

Reviewer #2: This paper introduces a new multiple instance fine-mapping method that can predict causal variants based on their underlying DNA sequences. The main validation approach is based on building polygenic risk scores across different ancestry groups and measuring their portability/transferability. The authors used 20 traits across 5 ancestries. The approach is based on training a machine learning model that uses weakly-labelled data. I believe the method is relevant and is very well described in the Methods section.

I don’t have major concerns but a few questions/clarifications and requests:

* Section 3.5 in the Results (“Syntax analysis of a MIFM trained model”) starts with what the authors did, but it is not clear why they are doing that. This paragraph should start with a sentence saying what is the question that the section/paragraph is trying to answer. The authors directly jump into what they did “We analyzed...”

* In Methods, line 227, in the text “... be the p-value for the k-th variant” maybe you meant “j-th variant”? (If I’m following correctly).

* In Methods, equations 4 and 5, when you define positive and negative bags, how do you handle cases where variants are borderline with respect to the threshold? Shouldn't you use a larger gap to ensure non-significant results? In the current definition, with a threshold of 0.05 (just as an example) a variant with p-value 0.04999 would be positive, and one with p-value 0.050001 would be negative. Could this be a potential problem?

* In Methods, under “Dataset and model training” could the authors explain more the reason for having a token “V”? Is it correct that, per bag, this indicates the variant of interest?

* There is a GitHub repository (https://github.com/HealthML/multiple-instance-fine-mapping) with the method and examples on how to make predictions using a pre-trained model. However, the code for reproducing the figures/tables is not available.

**********

**Have all data underlying the figures and results presented in the manuscript been provided?**

Large-scale datasets should be made available via a public repository as described in the *PLOS Genetics*  data availability policy, and numerical data that underlies graphs or summary statistics should be provided in spreadsheet form as supporting information.

Reviewer #1: Yes

Reviewer #2: **No:** There is a GitHub repository (https://github.com/HealthML/multiple-instance-fine-mapping) with the method and examples on how to make predictions using a pre-trained model. However, the code for reproducing the figures/tables is not available.

**********

PLOS authors have the option to publish the peer review history of their article (what does this mean?). If published, this will include your full peer review and any attached files.

If you choose “no”, your identity will remain anonymous but your review may still be made public.

**Do you want your identity to be public for this peer review?**  For information about this choice, including consent withdrawal, please see our Privacy Policy.

Reviewer #1: No

Reviewer #2: No

[NOTE: If reviewer comments were submitted as an attachment file, they will be attached to this email and accessible via the submission site. Please log into your account, locate the manuscript record, and check for the action link "View Attachments". If this link does not appear, there are no attachment files.]

**Figure resubmission:**

-->While revising your submission, we strongly recommend that you use PLOS’s NAAS tool (https://ngplosjournals.pagemajik.ai/artanalysis) to test your figure files. NAAS can convert your figure files to the TIFF file type and meet basic requirements (such as print size, resolution), or provide you with a report on issues that do not meet our requirements and that NAAS cannot fix.

After uploading your figures to PLOS’s NAAS tool - https://ngplosjournals.pagemajik.ai/artanalysis, NAAS will process the files provided and display the results in the "Uploaded Files" section of the page as the processing is complete. If the uploaded figures meet our requirements (or NAAS is able to fix the files to meet our requirements), the figure will be marked as "fixed" above. If NAAS is unable to fix the files, a red "failed" label will appear above. When NAAS has confirmed that the figure files meet our requirements, please download the file via the download option, and include these NAAS processed figure files when submitting your revised manuscript.

**Reproducibility:**

To enhance the reproducibility of your results, we recommend that authors of applicable studies deposit laboratory protocols in protocols.io, where a protocol can be assigned its own identifier (DOI) such that it can be cited independently in the future. Additionally, PLOS ONE offers an option to publish peer-reviewed clinical study protocols. Read more information on sharing protocols at https://plos.org/protocols?utm_medium=editorial-email&utm_source=authorletters&utm_campaign=protocols

# Author comment: Author response to Decision Letter 1

**DOI:** 10.1371/journal.pgen.1012208.r003

11 Mar 2026

Attachment Submitted filename: 16_03_2026_Response_to_Reviewers.pdf

# Peer review: Decision Letter 1

**DOI:** 10.1371/journal.pgen.1012208.r004

**Authors:** Heather Cordell, Xiaofeng Zhu, Heather Cordell, Xiaofeng Zhu

5 Jun 2026

Dear Dr Rakowski,

We are pleased to inform you that your manuscript entitled "Multiple instance fine-mapping: predicting causal regulatory variants with a deep sequence model" has been editorially accepted for publication in PLOS Genetics. Congratulations!

Before your submission can be formally accepted and sent to production you will need to complete our formatting changes, which you will receive in a follow up email. Please be aware that it may take several days for you to receive this email; during this time no action is required by you. Please note: the accept date on your published article will reflect the date of this provisional acceptance, but your manuscript will not be scheduled for publication until the required changes have been made.

Once your paper is formally accepted, an uncorrected proof of your manuscript will be published online ahead of the final version, unless you’ve already opted out via the online submission form. If, for any reason, you do not want an earlier version of your manuscript published online or are unsure if you have already indicated as such, please let the journal staff know immediately at plosgenetics@plos.org.

In the meantime, please log into Editorial Manager at https://www.editorialmanager.com/pgenetics/, click the "Update My Information" link at the top of the page, and update your user information to ensure an efficient production and billing process. Note that PLOS requires an ORCID iD for all corresponding authors. Therefore, please ensure that you have an ORCID iD and that it is validated in Editorial Manager. To do this, go to ‘Update my Information’ (in the upper left-hand corner of the main menu), and click on the Fetch/Validate link next to the ORCID field.  This will take you to the ORCID site and allow you to create a new iD or authenticate a pre-existing iD in Editorial Manager.

If you have a press-related query, or would like to know about making your underlying data available (as you will be aware, this is required for publication), please see the end of this email. If your institution or institutions have a press office, please notify them about your upcoming article at this point, to enable them to help maximise its impact. Inform journal staff as soon as possible if you are preparing a press release for your article and need a publication date.

Thank you again for supporting open-access publishing; we are looking forward to publishing your work in PLOS Genetics!

Yours sincerely,

Heather J Cordell

Academic Editor

PLOS Genetics

Xiaofeng Zhu

Section Editor

PLOS Genetics

Aimée Dudley

Editor-in-Chief

PLOS Genetics

Anne Goriely

Editor-in-Chief

PLOS Genetics

www.plosgenetics.org

BlueSky: @plos.bsky.social

----------------------------------------------------

Comments from the reviewers (if applicable):

Reviewer's Responses to Questions

**Comments to the Authors:**

**Please note here if the review is uploaded as an attachment.**

Reviewer #1: The authors have addressed my previous suggestions well. I have no further suggestions or concerns.

**********

**Have all data underlying the figures and results presented in the manuscript been provided?**

Large-scale datasets should be made available via a public repository as described in the *PLOS Genetics*  data availability policy, and numerical data that underlies graphs or summary statistics should be provided in spreadsheet form as supporting information.

Reviewer #1: Yes

**********

PLOS authors have the option to publish the peer review history of their article (what does this mean?). If published, this will include your full peer review and any attached files.

If you choose “no”, your identity will remain anonymous but your review may still be made public.

**Do you want your identity to be public for this peer review?**  For information about this choice, including consent withdrawal, please see our Privacy Policy.

Reviewer #1: No

----------------------------------------------------

**Data Deposition**

If you have submitted a Research Article or Front Matter that has associated data that are not suitable for deposition in a subject-specific public repository (such as GenBank or ArrayExpress), one way to make that data available is to deposit it in the Dryad Digital Repository. As you may recall, we ask all authors to agree to make data available; this is one way to achieve that. A full list of recommended repositories can be found on our website.

The following link will take you to the Dryad record for your article, so you won't have to re‐enter its bibliographic information, and can upload your files directly:

http://datadryad.org/submit?journalID=pgenetics&manu=PGENETICS-D-25-00787R1

More information about depositing data in Dryad is available at http://www.datadryad.org/depositing. If you experience any difficulties in submitting your data, please contact help@datadryad.org for support.

Additionally, please be aware that our data availability policy requires that all numerical data underlying display items are included with the submission, and you will need to provide this before we can formally accept your manuscript, if not already present.

----------------------------------------------------

**Press Queries**

If you or your institution will be preparing press materials for this manuscript, or if you need to know your paper's publication date for media purposes, please inform the journal staff as soon as possible so that your submission can be scheduled accordingly. Your manuscript will remain under a strict press embargo until the publication date and time. This means an early version of your manuscript will not be published ahead of your final version. PLOS Genetics may also choose to issue a press release for your article. If there's anything the journal should know or you'd like more information, please get in touch via plosgenetics@plos.org.

# Editor report: Acceptance letter

**DOI:** 10.1371/journal.pgen.1012208.r005

**Authors:** Heather Cordell, Xiaofeng Zhu

PGENETICS-D-25-00787R1

Multiple instance fine-mapping: predicting causal regulatory variants with a deep sequence model

Dear Dr Rakowski,

We are pleased to inform you that your manuscript entitled "Multiple instance fine-mapping: predicting causal regulatory variants with a deep sequence model" has been formally accepted for publication in PLOS Genetics! Your manuscript is now with our production department and you will be notified of the publication date in due course.

The corresponding author will soon be receiving a typeset proof for review, to ensure errors have not been introduced during production. Please review the PDF proof of your manuscript carefully, as this is the last chance to correct any errors. Please note that major changes, or those which affect the scientific understanding of the work, will likely cause delays to the publication date of your manuscript.

Soon after your final files are uploaded, unless you have opted out or your manuscript is a front-matter piece, the early version of your manuscript will be published online. The date of the early version will be your article's publication date. The final article will be published to the same URL, and all versions of the paper will be accessible to readers.

For Research Articles, you will receive an invoice from PLOS for your publication fee after your manuscript has reached the completed accept phase. If you receive an email requesting payment before acceptance or for any other service, this may be a phishing scheme. Learn how to identify phishing emails and protect your accounts at https://explore.plos.org/phishing.

Thank you again for supporting PLOS Genetics and open-access publishing. We are looking forward to publishing your work!

With kind regards,

Anitha Samidurai

PLOS Genetics

On behalf of:

The PLOS Genetics Team

Carlyle House, Carlyle Road, Cambridge CB4 3DN | United Kingdom

plosgenetics@plos.org | +44 (0) 1223-442823

plosgenetics.org | Twitter: @PLOSGenetics
